# Functional ProTracer identifies patterns of cell proliferation in tissues and underlying regulatory mechanisms

**DOI:** 10.1038/s41536-023-00318-y

**Published:** 2023-08-03

**Authors:** Xiuxiu Liu, Maoying Han, Wendong Weng, Yan Li, Wenjuan Pu, Kuo Liu, Xufeng Li, Lingjuan He, Ruilin Sun, Ruling Shen, Yulong He, Dandan Liang, Yi-Han Chen, Qing-Dong Wang, Jan S. Tchorz, Bin Zhou

**Affiliations:** 1 New Cornerstone Science Laboratory, State Key Laboratory of Cell Biology, Shanghai Institute of Biochemistry and Cell Biology, Center for Excellence in Molecular Cell Science, Chinese Academy of Sciences, University of Chinese Academy of Sciences, Shanghai, China; 2 Shandong Laboratory of Yantai Drug Discovery, Bohai Rim Advanced Research Institute for Drug Discovery, Yantai, Shandong China; 3 State Key Laboratory of Drug Research, Shanghai Institute of Materia Medica, Chinese Academy of Sciences, Shanghai, China; 4 Key Laboratory of Systems Health Science of Zhejiang Province, School of Life Science, Hangzhou Institute for Advanced Study, University of Chinese Academy of Sciences, Hangzhou, China; 5 Westlake University School of Life Sciences, Hangzhou, China; 6 Shanghai Model Organisms Center, Inc., Shanghai, China; 7 Shanghai Laboratory Animal Research Center, Shanghai, China; 8 Cyrus Tang Hematology Center, Collaborative Innovation Center of Hematology, State Key Laboratory of Radiation Medicine and Protection, Cam-Su Genomic Resources Center, Soochow University, Suzhou, China; 9 State Key Laboratory of Cardiology, Shanghai East Hospital, Tongji University School of Medicine, Shanghai, China; 10 Bioscience Cardiovascular, Research and Early Development, Cardiovascular, Renal and Metabolism (CVRM), BioPharmaceuticals R&D, AstraZeneca, Gothenburg, Sweden; 11 Novartis Institutes for BioMedical Research, Novartis Pharma AG, Basel, Switzerland; 12 School of Life Science and Technology, ShanghaiTech University, Shanghai, China

**Keywords:** Cytokinesis, Self-renewal

## Abstract

A genetic system, ProTracer, has been recently developed to record cell proliferation in vivo. However, the ProTracer is initiated by an infrequently used recombinase Dre, which limits its broad application for functional studies employing floxed gene alleles. Here we generated Cre-activated functional ProTracer (fProTracer) mice, which enable simultaneous recording of cell proliferation and tissue-specific gene deletion, facilitating broad functional analysis of cell proliferation by any Cre driver.

The ability to experimentally measure cell proliferation is the basis for understanding the sources of the cells that drive organ development, tissue homeostasis, and regeneration. Recently, we developed a genetic model named ProTracer to enable long-term continuous recording of cell proliferation in vivo^1^. ProTracer is initiated by Dre-rox recombination, which releases *Ki67-Cre* to prime the genetic system for recording cell proliferation over time (Fig. 1a). In spite of its initial application in uncovering regional hepatocyte or cardiomyocyte generation patterns in tissue homeostasis and repair^1,2^, the Dre-activated ProTracer has two limitations that prevent its broad application across biomedical research fields. First, Dre is an infrequently used recombinase in contrast with the widely used Cre recombinase. The lack of Dre mouse lines makes it cumbersome to use ProTracer for studying tissue-specific cell proliferation. Second, gene deletion-based functional analysis of cell proliferation was not possible with ProTracer, since all gene alleles allowing conditional deletion are *loxP*-flanked (floxed) rather than *rox*-flanked. To overcome the above limitations, we designed a new version of ProTracer in which Cre-*loxP* recombination initiates this recording of cell proliferation and therefore enables simultaneous conditional deletion of any floxed genes (named functional ProTracer, fProTracer in short, Fig. 1b). Our new Cre-induced fProTracer, therefore, combines cell recording features with full compatibility to the pre-existing rich resources of Cre and floxed mouse lines.

**Fig. 1:**
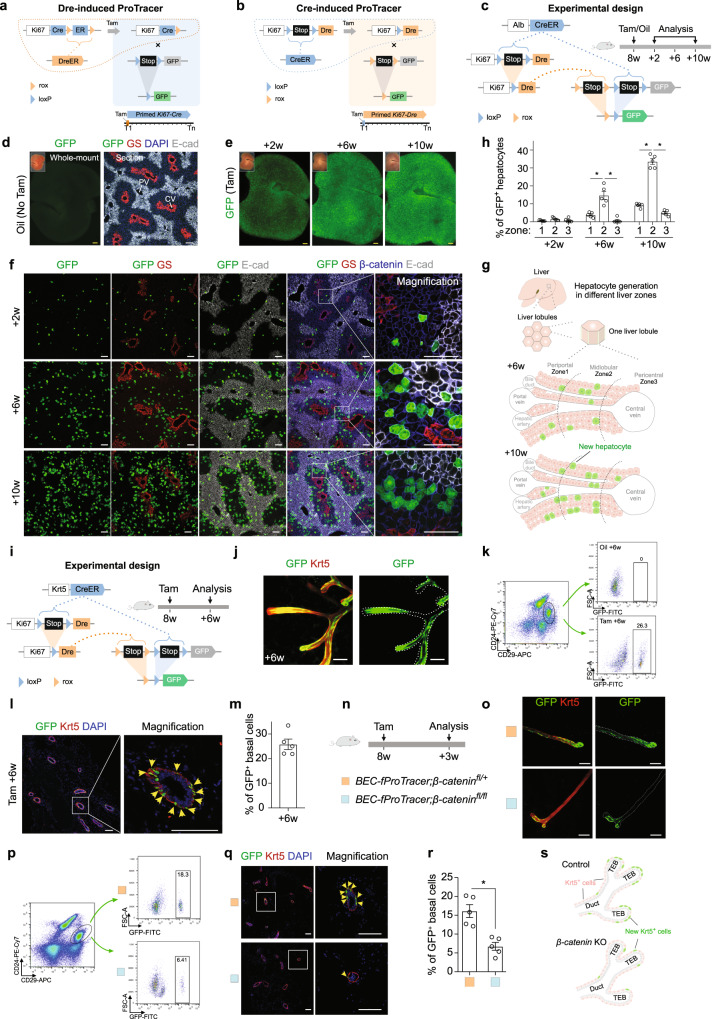
Generation and characterization of Cre-induced ProTracer system. **a**, **b** Schematic diagrams showing Dre-induced ProTracer (**a**) and Cre-induced fProTracer (**b**) for the genetic recording of cell proliferation. **c** Schematic diagram showing the experimental design for *Alb-CreER* induced fProTracer. Tam, tamoxifen. **d** Whole-mount liver images and immunostaining of liver sections of mice without Tam. **e** Whole-mount fluorescence images of livers collected at 2w, 6w, and 10w after Tam. **f** Immunostaining for GFP, GS, β-catenin, and E-cad on liver sections. **g** A cartoon image showing new hepatocyte generation highly enriched in zone 2. **h** Quantification of the percentage of GFP^+^ hepatocytes in each zone. Data were mean ± SEM; *P < 0.05. **i** A schematic diagram showing the experimental design for *Krt5-CreER* induced fProTracer. **j** Whole-mount fluorescent staining for GFP and Krt5 of mammary glands. **k** Flow cytometric analysis of GFP^+^ basal cells in mammary gland from mice treated with or without Tam. **l** Immunostaining for GFP and Krt5 on mammary tissue sections. Arrowheads, GFP^+^Krt5^+^ cells. **m** Quantification of the percentage of basal cells expressing GFP. Data were mean ± SEM; *n* = 5. **n** A schematic diagram showing the experimental design. BEC, basal epithelial cells. **o** Whole-mount fluorescent images of mammary glands. **p** Flow cytometric analysis of GFP^+^ basal cells from the mutant and control mice. **q** Immunostaining for Krt5 and GFP on mammary tissue sections. **r** Quantification of the percentage of GFP^+^ basal cells in mice at 3 weeks post Tam. Data were mean ± SEM; *P < 0.05. **s** A cartoon image shows the proliferation pattern of Krt5^+^ BECs in the mammary gland. Scale bars: white,100 μm; yellow, 1 mm. Each image is representative of five individual mouse samples.

To develop fProTracer, we first generated two mouse lines: *Ki67-L-Dre* (*Ki67-loxP-Stop-loxP-IRES-Dre*, Supplementary Fig. 1a–c) and *R26-RL-GFP* (*Rosa26-rox-Stop-rox-loxP-Stop-loxP-GFP*, Supplementary Fig. 2). We crossed the *Ki67-L-Dre* line with the inducible Cre driver *UBC-CreER*^3^ and rox reporter *R26-R-tdT* (*R26-rox-Stop-rox-tdTomato*)^4^, yielding *UBC-CreER;Ki67-L-Dre;R26-R-tdT* mice. After tamoxifen (Tam) treatment, we detected tdTomato (tdT)^+^ cells in *UBC-CreER;Ki67-L-Dre;R26-R-tdT* embryos, demonstrating Cre-induced Dre expression from *Ki67* allele could activate the genetic reporter (Supplementary Fig. 1d–g). No detectable tdT^+^ cells in *Ki67-L-Dre;R26-R-tdT* mice after Tam treatment excluded leakiness of *Ki67-L-Dre;R26-R-tdT* mice (Supplementary Fig. 1h). For *R26-RL-GFP* knock-in mice, we found that GFP was only activated in cells that expressed both Cre and Dre (Supplementary Fig. 2). In the fProTracer system, *Ki67-L-Dre*;*R26-RL-GFP* were used to respond to Cre to trace cell proliferation in a tissue-specific manner.

Our previous study using ProTracer reported highly regional hepatocyte proliferation during liver homeostasis^1^. To examine whether fProTracer uncovers a similar patterning of hepatocyte generation, we crossed a hepatocyte-specific Cre line, *Alb-CreER* (Supplementary Fig. 3a–d), with *Ki67-L-Dre;R26-RL-GFP* mice (Fig. 1c). In these hepatocyte-specific fProTracer mice, Tam-induced Cre-*loxP* recombination removes *loxP-Stop-loxP* in both *Ki67-L-Dre* and *R26-RL-GFP* alleles, yielding two new alleles in hepatocytes, *Ki67-Dre* and *R26-R-GFP*, respectively (Fig. 1c). When hepatocytes proliferate, Dre-*rox* recombination removes the *rox*-flanked Stop sequence, leading to permanent GFP expression as an indicator of hepatocyte proliferation (Fig. 1c). There were barely any GFP signals in hepatocytes of *Alb-CreER;Ki67-L-Dre;R26-RL-GFP* mice without Tam (Fig. 1d) or in A*lb-CreER;R26-RL-GFP* mice treated with Tam (Supplementary Fig. 3e–h). In *Alb-CreER;Ki67-L-Dre;R26-RL-GFP* mice treated with Tam, increased GFP signals were shown by whole-mount fluorescent or tissue staining images from 2 weeks to 10 weeks after Tam (Fig. 1e, f). Quantification of the percentage of GFP^+^ hepatocytes in each liver zones revealed a significantly higher percentage of hepatocytes expressing GFP in zone 2 compared with those in zone 1 or 3 at all time points examined (Fig. 1g, h), highlighting preferential mid-lobular hepatocyte generation, consistent with the previous results^1,5,6^.

To showcase fProTracer by another example, we crossed a basal epithelial cell (BEC)-specific Cre, *Krt5-CreER* (Supplementary Fig. 4a–c), with our *Ki67-L-Dre;R26-RL-GFP* mice (Fig. 1i). We treated adult *Krt5-CreER;Ki67-L-Dre;R26-RL-GFP* (*BEC-fProTracer*) mice with Tam and collected mammary glands for analysis at 6 weeks after Tam treatment (Fig. 1i). Whole-mount fluorescence staining for GFP and Krt5 showed that GFP^+^ basal cells were highly enriched at the terminal end buds (TEB, Fig. 1j), suggesting that epithelial cells in the terminal end bud undergo more rapid growth than other regions in the mammary gland. Flow cytometric analysis of CD29^Hi^CD24^+^ basal cells revealed about 26% basal cells expressing GFP at 6 weeks post Tam treatment, while no detectable GFP cells were found in oil-treated mice (Fig. 1k). Immunostaining for GFP and Krt5 on mammary tissue sections revealed about 25% of GFP^+^Krt5^+^ basal cells were labeled 6 weeks after Tam treatment (Fig. 1l, m). Krt5^+^GFP^+^ cells were also detected in other tissues (Supplementary Fig. 4d), while no detectable GFP^+^ cells were shown in tissues collected from *Krt5-CreER;Ki67-L-Dre;R26-RL-GFP* mice without Tam or from *Krt5-CreER;R26-RL-GFP* mice with Tam treatment (Supplementary Fig. 4e–h).

Having demonstrated the cell proliferation recording capability of fProTracer, we next evaluated the Cre-mediated gene deletion enabling simultaneous functional analysis of the underlying regulatory mechanisms. WNT signaling acts as an important morphogenetic signaling pathway and plays a crucial role in the differentiation, growth, and morphogenesis during mammary gland development^7–9^. We, therefore, examined the role of WNT signaling in regulating mammary basal cell proliferation during homeostasis. To specifically delete *β-catenin* in BECs, we crossed *BEC-fProTracer* with floxed *β-catenin* mice (*β-catenin*^*fl*^)^10^ to generate *BEC-fProTracer*;*β-catenin*^*fl/fl*^ (mutant) mice and used *BEC-fProTracer*;*β-catenin*^*fl/+*^ (control) mice as internal controls (Fig. 1n). We collected mammary glands to analyze GFP labeling at 3 weeks post Tam (Fig. 1n). We found a significant reduction in GFP^+^ basal cells in the mammary glands of mutant mice compared with those in control mice (Fig. 1o–r). Whole-mount fluorescence staining of GFP and Krt5 revealed a noticeable reduction of GFP^+^ basal cells in both duct and TEB regions (Fig. 1o). The decrease in GFP^+^ basal cells was confirmed by flow cytometric analysis (Fig. 1p and Supplementary Fig. 4i). These data suggest that β-catenin is required for basal cell proliferation during mammary gland homeostasis (Fig. 1s).

Given the important function of lymphatic vessels in homeostasis, disease, and regeneration^11^, we now studied the cellular and molecular mechanisms regulating the proliferation of lymphatic endothelial cells (LECs). We crossed LEC-specific Cre, *Prox1-CreER*^12^, with our *Ki67-L-Dre;R26-RL-GFP* mice, and also with a *R26-L-tdT*^13^ reporter to facilitate visualizing lymphatic vessels from whole-mount fluorescence imaging (Fig. 2a). We collected mesentery to study lymphatic vessels from *Prox1-CreER;R26-L-tdT;Ki67-L-Dre;R26-RL-GFP* (LEC-fProTracer) mice at 6 and 12 weeks after Tam treatment (Fig. 2b, c). Whole-mount fluorescence images showed that GFP^+^ cells expressed both Prox1 and tdT, and these GFP^+^ LECs were highly restricted in the valves rather than collecting vessels while no detectable GFP^+^ cells were shown in mice without Tam (Fig. 2d–g). We found increased GFP^+^ lymphatic ECs in both collecting vessels and valves from 6 weeks to 12 weeks after Tam treatment (Fig. 2f). GFP^+^ LECs were also detected in lymphatic vessels in other tissues (Supplementary Fig. 5a–d). As technical controls, we detected rare GFP^+^ cells in tissues collected from LEC-fProTracer mice without Tam (Supplementary Fig. 5e) or from *Prox1-CreER;R26-RL-GFP* mice with Tam (Supplementary Fig. 5f–i). These data demonstrated the ability of fProTracer for the genetic recording of lymphatic EC proliferation over time, which was highly enriched in valves (Fig. 2g).

**Fig. 2:**
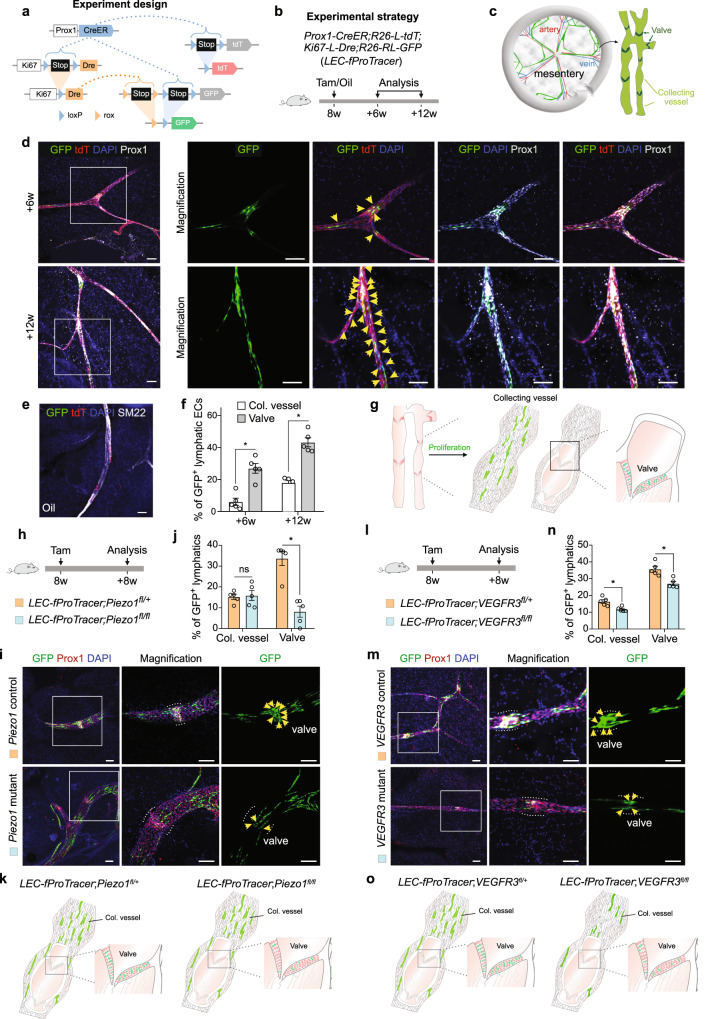
Functional analysis of lymphatic EC generation by Cre-induced fProTracer. **a**, **b** Schematic diagrams showing the experimental design for *Prox1-CreER* induced fProTracer. GFP denotes cell proliferation and tdT marks lymphatic ECs. **c** A cartoon image showing valves and collecting vessels of mesentery lymphatics. **d** Whole-mount fluorescent staining for GFP, tdT, and Prox1 of mesentery lymphatics. Arrowheads, GFP^+^tdT^+^ lymphatic ECs, which are enriched in valve regions. **e** No GFP^+^ ECs are detected in lymphatics collected from mice without Tam. **f** Quantification of lymphatic ECs expressing GFP in collecting (col.) vessels and valves collected at 6w and 12w after Tam. Data were mean ± SEM; n = 5; *P < 0.05. **g** A cartoon image showing the generation of new lymphatic ECs in col. vessels and valves. **h**, **l** A schematic diagram showing the experimental design for studying functions of Piezo1 (**h**) and VEGFR3 (**k**) in regulating LEC proliferation. **i**, **m** Whole-mount fluorescent staining for GFP and Prox1 on mutant and control mouse samples. Arrowheads, GFP^+^ lymphatic ECs in valves. **j**, **n** Quantification of the lymphatic ECs expressing GFP in valves and col. vessels in mutant and control mouse samples. **k**, **o** Cartoon images, the proliferation of LECs is regulated by *Piezo1* and *Vegfr3*. Data were mean ± SEM; *n* = 5; *P < 0.05; ns not significant. Each image is representative of five individual mouse samples.

Having demonstrated the cell proliferation recording capability of fProTracer in LECs, we next evaluated the Cre-mediated gene deletion enabling simultaneous functional analysis. Recent studies reported that Piezo1 was expressed in LECs and Piezo1-regulated mechanotransduction controls flow-activated LEC expansion during development^14,15^, suggesting its potential role in regulating lymphatic maintenance. We analyzed the Piezo1 expression in the lymphatics vessels and found that Piezo1 was broadly expressed in LECs and especially enriched in the valve regions (Supplementary Fig. 6). However, the role of Piezo1 in LEC proliferation during homeostasis remained elusive. To investigate the potential function of Piezo1 in regulating LEC proliferation, we generated *LEC-fProTracer;Piezo1*^*fl/fl*^ mice (mutant) and *LEC-fProTracer;Piezo1*^*fl/+*^ mice (control), and collected mesentery for analysis at 8 weeks after Tam treatment (Fig. 2h). We found a significant reduction of GFP^+^ LECs in the valves of the mutant compared with control mice. However, we did not find differences in collecting vessels of both groups (Fig. 2i–k), suggesting that Piezo1 is required for LEC proliferation in valves rather than collecting vessels. This regional proliferation pattern suggests that LECs in the valve region could be more susceptible to daily wear and tear, and the regional proliferation pattern in valve regions was controlled by Peizo1. How the external mechanical force is transduced into internal molecular signals that trigger cell proliferation merits further investigation in the future. Moreover, we next conditionally deleted *VEGFR3* using *LEC-fProTracer;Vegfr3*^*fl/fl*^ mice to study the role of VEGFR3 in LEC proliferation during homeostasis (Fig. 2l), as VEGFR3 has been known to promote lymphangiogenesis during development^16^. Simultaneous proliferation recording and *VEGFR3* deletion in lymphatics showed impaired LEC proliferation in the valve or collecting vessels (Fig. 2m–o). These data suggested that LEC generation in different compartments of lymphatics was distinctly regulated by unique gene programs.

This study reported a Cre-activated fProTracer for broad application of in vivo cell proliferation recording with simultaneous functional analysis of genes that potentially regulate cell proliferation. By using three Cre drivers that target endoderm, ectoderm, and mesoderm cell lineages, respectively, we demonstrated efficient and specific recording of in vivo cell proliferation. These data not only validated the enriched mid-lobular zone 2 hepatocyte proliferation but also revealed the interesting observations of highly regional cell proliferation in mammary terminal end buds and lymphatic valves during homeostasis. Genetic dissection of gene programs that regulate cell proliferation is also feasible with fProTracer, which is now adaptable and compatible with abundant Cre drivers and floxed allele resources. The fProTracer system can also be applied to other gene manipulation elements, such as the over-expression of specific transgenes by e.g. *loxP*-stop-*loxP*-transgene alleles. Furthermore, fProTracer could also be used to study the effects of non-genetic materials or reagents on in vivo cell proliferation. Our fProTracer system has its limitation in combination with other adaptations like the mosaic analysis with double markers (MADM) system, as fProTracer relies on Dre recombinase to label cells and the recombination efficiency would be low, especially for inter-chromosome recombination in the MADM system. Moreover, fProTracer is compatible with our recently developed dual recombinase-responding confetti reporter^17^ for clonal study simultaneously with functional analysis. Above all, the application of Cre-activated fProTracer has the potential to greatly accelerate the mechanistic and phenotypic dissection of cell proliferation mechanisms in tissue homeostasis, regeneration, and also disease.

## Methods

### Mice

All mice were used in accordance with the guidelines of the Institutional Animal Care and Use Committee of the Center for Excellence in Molecular Cell Science, Shanghai Institute of Biochemistry and Cell Biology, Chinese Academy of Sciences. All animal experiments were performed according to the protocols which were approved by the Institutional Animal Care and Use Committee of the Center for Excellence in Molecular Cell Science, Shanghai Institute of Biochemistry and Cell Biology, Chinese Academy of Sciences. Mouse lines *Prox1-CreER*, *β-catenin*^*fl/+*^, *Piezo1*^*fl/+*^, *VEGFR3*^*fl/+*^, *UBC-CreER*, *R26-R-tdT*, *Tnni3-Dre*, *ACTB-Cre*, *CAG-Dre*, *R26-L-tdT*, and *Piezo1-CreER* were generated or mentioned before^3,10,12,16,18–21^. New knock-in mouse lines *Ki67-L-Dre*, *R26-RL-GFP*, *Alb-CreER*, and *Krt5-CreER* were generated by homologous recombination using CRISPR/Cas9 technology. These new mouse lines were generated by the Shanghai Model Organisms Center, Inc. (SMOC). These mice were bred in a C57BL6/ICR mixed background. CreER or DreER require Tam (the metabolite of Tam binds to ER) to access the nucleus to identify the lox*P* or *rox* sites. Tam dissolved in corn oil was given to mice at a dosage of 0.2 mg/g mouse body weight. *Krt5-CreER* has a strong CreER activity, so 0.1 mg/g mouse body weight of Tam was injected into *Krt5-CreER* mice specifically to avoid the potential cross-talk between *Krt5-CreER* and rox sites in *R26-RL-GFP*. Each experiment was repeated by five individual mice and the figures shown were representative of the five samples.

### Genomic PCR

Genomic DNA was prepared from the mouse toes or embryo tails. Tissues were lysed by lysis buffer (100 mM Tris-HCl, 5 mM EDTA, 0.2% SDS, 200 mM NaCl, and 100 µg/mL Proteinase K) at 55 °C overnight. About 750 µL pure ethanol was added to the lysis mixture and mixed thoroughly, followed by centrifugation at maximum speed for 5 min at room temperature to collect the DNA samples. Then the supernatant was discarded and the mixture was dried at room temperature. About 100–200 µL double-distilled H_2_O was added to dissolve the DNA. The genomic PCR primer pairs were designed for the mutant alleles spanning both endogenous genomic fragments and insert fragments. All the genomic PCR primer sequences were listed in Supplementary Table 1.

### Whole-mount imaging and sectioning

The tissue samples were fixed with 4% paraformaldehyde (PFA) for 30 min to 1 h according to the sample size, followed by washing with PBS three times. The fixed samples were placed into an agarose filled petri-dish for bright-field and fluorescence imaging by a Zeiss stereoscopic microscope (AxioZoom V16). For cryo-sections, tissues were sectioned to slides of 10-µm thickness after dehydration by 30% sucrose (wt/vol in PBS) overnight and pre-embedding with OCT (Sakura) at 4 °C for 1 h.

### Mouse bone decalcifying

Mouse bones were decalcified with a previously reported method with little modification^22^. Femurs and tibias were fixed in 4% PFA at 4 °C overnight after clearing the muscle away. After washing with PBS, the femurs and tibias were incubated with 10% (wt/vol) sucrose and 10% (wt/vol) EDTA in PBS at 4 °C for several days. We decalcified the adult mouse bones for 7 days. The decalcified bones were embedded with OCT compound for further sectioning and analysis.

### Immunostaining

Immunostaining was performed as previously described in ref. ^2^. Tissue sections were blocked with 2.5% normal donkey serum dissolved in 0.2% PBST (0.2% (vol/vol) Triton X-100 dissolved in PBS) after washing with PBS three times. The tissue sections were incubated with primary antibody diluted in 0.2% PBST at 4 °C overnight. On the next day, sections were incubated with secondary antibodies diluted in 0.2% PBST at room temperature for 30 min, followed by PBS washing three times. The slides were washed with PBS three times. The slides were mounted with a mounting medium (Vector Lab). For weak signals, the endogenous peroxidase activity was quenched before blocking. Horseradish peroxidase or biotin-conjugated secondary antibodies and a tyramide signal amplification kit (PerkinElmer) were used after incubating the primary antibodies. For primary antibodies of murine origin, mouse immunoglobulins were blocked with an anti-mouse Fab antibody. The included primary antibodies are listed as follows: GFP (Nacalai tesque, 04404–84, 1:500), GS (Abcam, ab49873, 1:1000), β-catenin (BD Pharmingen, 610153, 1:200), E-cad (R&D, AF748, 1:500), Krt5 (Covance, 905504, 1:500), Prox1 (Abcam, ab101851), tdTomato (Rockland, 600–401–379, 1:1000), PECAM (BD Pharmingen, 553370, 1:500), TNNI3 (Abcam, ab56357, 1:200), HNF4α (Cell signaling, 3113 s, 1:1000), CK19 (Developmental Studies Hybridoma Bank, TROMA-III, 1:500), Desmin (R&D, AF3844, 1:100), VE-cad (R&D, AF1002, 1:100), and LYVE1 (eBioscience, 53–0443–80, 1:250). The corresponding secondary antibodies (JIR or Abcam) were diluted according to the instructions. Images were captured by using a Nikon confocal (Nikon A1 FLIM) or a Zeiss confocal (Zeiss LSM880), and captured images were analyzed by Image J (NIH) software.

### Clearing and Z-stack imaging

The mammary gland and mesenteric lymphatics were cleared by FUnGI clearing agent^23^. In brief, tissues were fixed with 4% PFA for 1 h and washed with PBST three times. Then tissues were incubated with primary antibodies for 4–5 days and then secondary antibodies for 4–5 days. Tissues were washed with PBS and cleared with FUnGI agent (50% glycerol (vol/vol), 2.5 M fructose, 2.5 M urea, 10.6 mM Tris Base, 1 mM EDTA) on a rotor at room temperature for 1–2 h. The tissues were mounted with the clearing agent. The consecutive Z-stack confocal images were obtained from the Nikon confocal or the Zeiss confocal as mentioned before, and the obtained images were analyzed by Image J (NIH) software.

### FACS

Mammary cells were isolated as described before^24^. Briefly, the third and fourth mammary glands (without lymphatic nodes) were collected and minced into small pieces. Then the samples were digested with an enzyme mix (5% fetal bovine serum, 1% penicillin-streptomycin-glutamine, 25 mM HEPES, 3000 U collagenase III (Worthington) dissolved in RPMI 1640 or DMEM) on a rotor at 120 rpm at 37 °C for 90 min. The mix was centrifuged at 1000 rpm for 5 min to discard the supernatant, which contained fatty tissue. The collected sediment was treated with red blood cell lysis buffer (eBioscience) at room temperature for 5 min. The incubation was stopped with Hank’s Balanced Salt Solution (HBSS) and the sample was centrifuged at 1000 rpm for 4 min to collect the tissues. The tissues were further digested with 0.25% trypsin-EDTA (Invitrogen) at 37 °C for 5 min and the digestion was stopped with the addition of DMEM and 0.1 mg/mL DNase I (Worthington) for a further 5 min. The single-cell suspension was collected by filtering through 70 µm cell strainers. Then cells were incubated with Fc block at room temperature for 5 min. After that, primary antibodies were added to the incubation mix for labeling the mammary cells at 4 °C for 30 min. After washing with HBSS solution, cells were re-suspended with HBSS containing DAPI before analyzing with Thermo Attune NxT. The FACS data were further analyzed by FlowJo (Tree Star) software. The included antibodies in this study are listed here: CD31 (Thermo Fisher Scientific, 48-0311-82, 1:100), CD45 (Thermo Fisher Scientific, 48-0451-82, 1:100), Ter-119 (Thermo Fisher Scientific, 48-5921-82, 1:100), CD24 (Biolegend, 101822, 1:100), CD29 (eBioscience, 17-0291-80, 1:100).

### Statistical analysis

All data were obtained from five individual biological samples unless specific otherwise. Data are represented as mean values ± s.e.m. An unpaired Student’s *t*-test was performed for data comparisons between two groups, while one-way ANOVA was performed for multiple-group comparisons. *P* < 0.05 is regarded as statistically significant.

### Reporting summary

Further information on research design is available in the Nature Research Reporting Summary linked to this article.

### Supplementary information

Supplementary information

Reporting Summary

## Data Availability

All data included in this study are included in the manuscript. The raw data are available upon request.
