# Supplementary material for: Functional ProTracer identifies patterns of cell proliferation in tissues and underlying regulatory mechanisms
Source: NPJ Regen Med. 2023 Aug 3;8:41. doi: 10.1038/s41536-023-00318-y (PMC10400583; doi:10.1038/s41536-023-00318-y)
Supplement: Supplementary file 1 — Supplementary information [file 41536_2023_318_MOESM1_ESM.pdf]

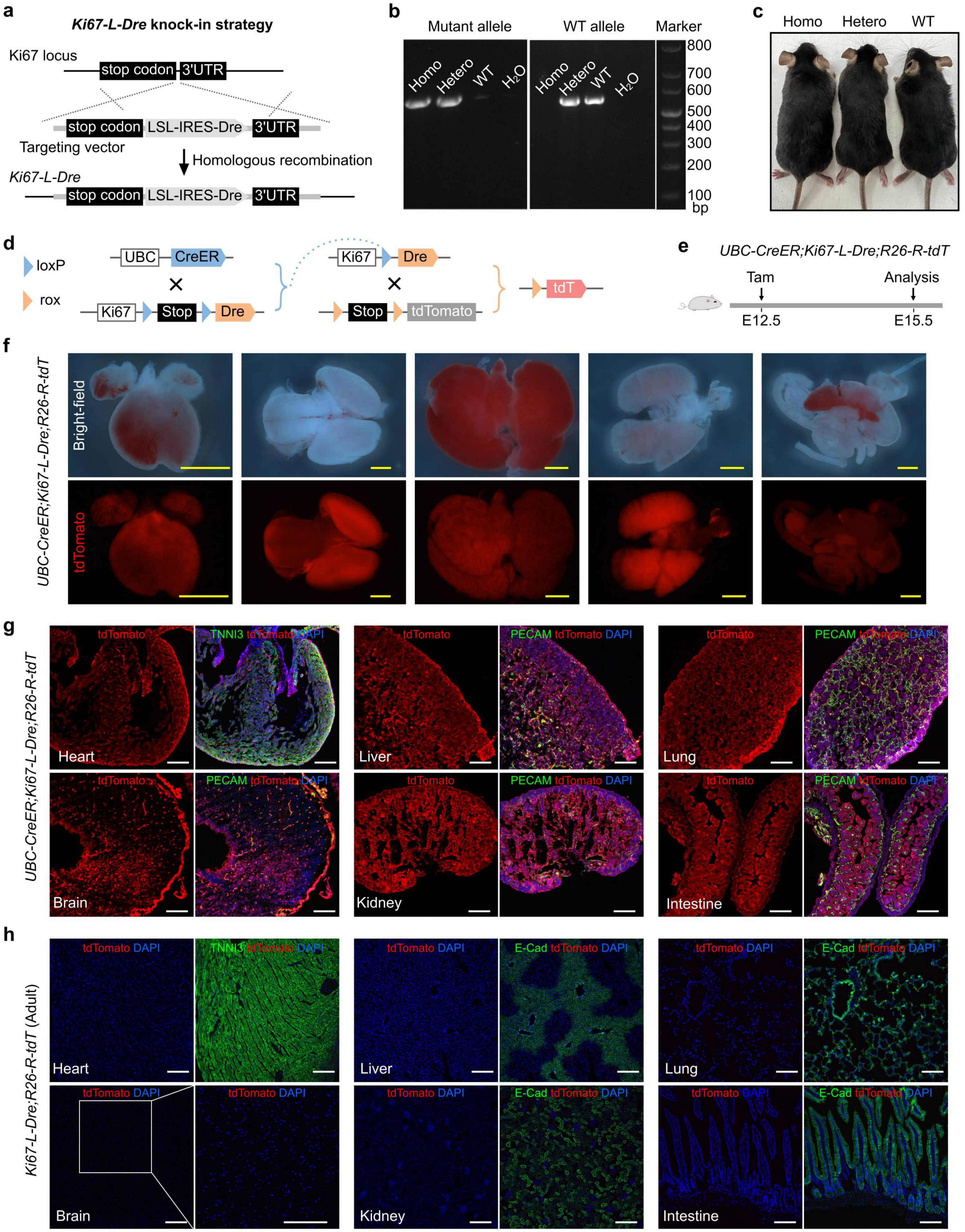

Supplementary Figure 1. Generation and characterization of *Ki67-L-Dre* mice.
a Schematic diagram showing the knock-in strategy for the generation of *Ki67-L-Dre* mice. **b** Genotyping results of *Ki67-L-Dre* homozygous (homo), heterozygous (hetero), and wild-type (WT) mice. The gels were processed in parallel. **c** Images of mice from the three genotype. The normal mouse status indicated that inserting "L-Dre" sequence into the *Ki67* locus didn't alert the endogenous *Ki67* gene activity. **d** Schematic diagram showing the strategy for cell proliferation recording using *UBC-CreER;Ki67-L-Dre;R26-R-tdT* mice. **e** Diagram of the experimental design. **f** Whole-mount bright-field and fluorescence images of tissues or organs collected from Tam-treated embryos. **g** Immunostaining for tdTomato and PECAM on tissue sections collected from Tam-treated embryos. **h** Immunostaning of tissue sections collected from Tam-treated adult *Ki67-L-Dre;R26-R-tdT* mice. Scale bars: yellow, 1 mm; white, 200 μm.

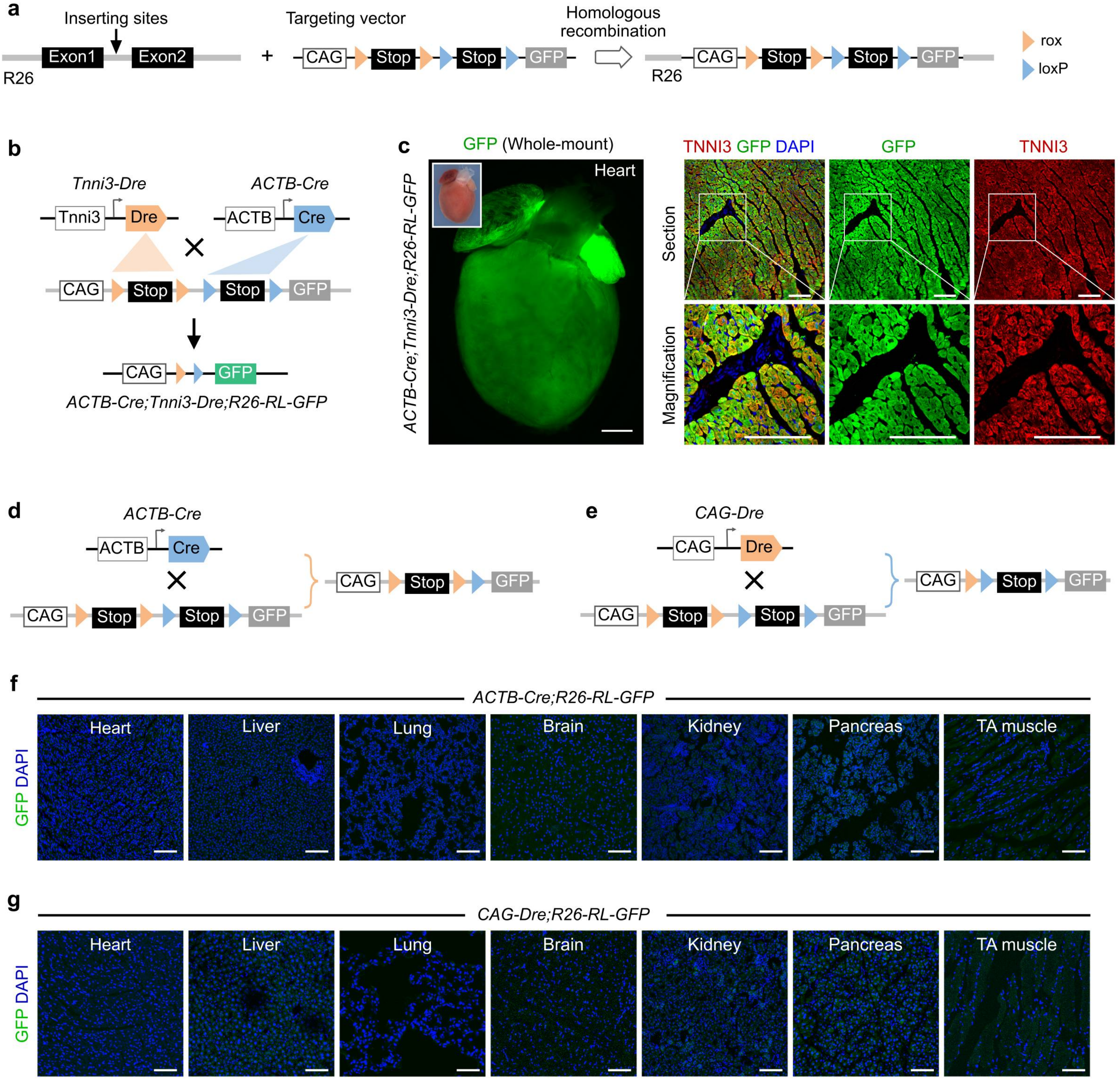

Supplementary Figure 2. Generation and characterization of *R26-RL-GFP* mice.
a Schematic diagram showing the the knock-in strategy for the generation of *R26-RL-GFP* mice. **b** Schematic diagram showing dual recombinations-induced GFP expression in *ACTB-Cre* and *Tnni3-Dre;R26-RL-GFP* mice. **c** Whole-mount bright-field (insert) and fluorescence images of the heart collected from 2 weeks' old *ACTB-Cre;Tnni3-Dre;R26-RL-GFP* mouse (left). Right panel shows immunostaining for TNNI3 and GFP on heart sections. **d**-**e** Schematic diagram showing the crossing of *ACTB-Cre* (d) or *CAG-Dre* (e) with *R26-RL-GFP*. **f**-**g** Immunostaining for GFP on *ACTB-Cre;R26-RL-GFP* (f) or *CAG-Dre;R26-RL-GFP* (g) tissue sections shows rare GFP^{+} cells in tissue sections. Scale bars,100 μm.

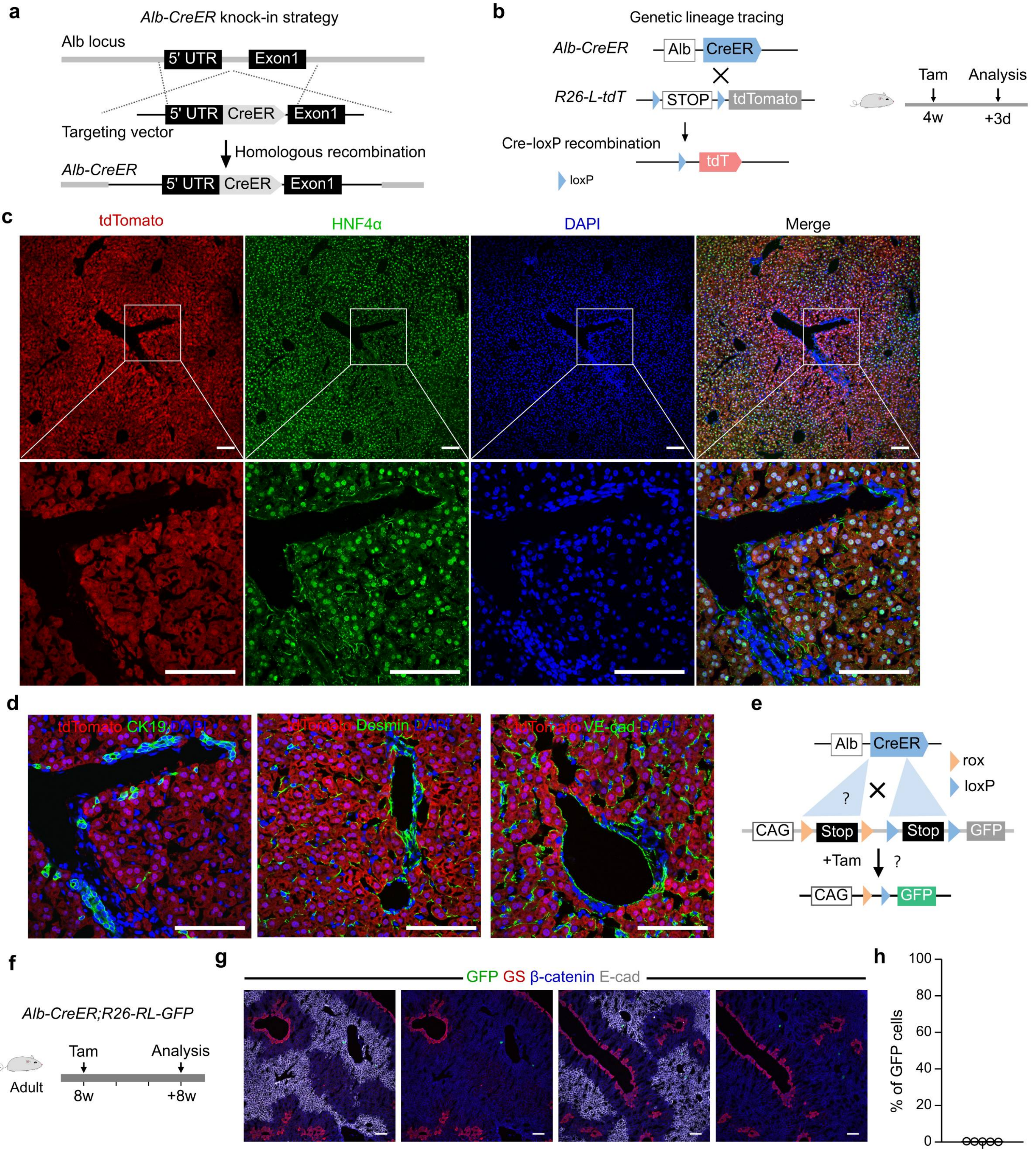

Supplementary Figure 3. Generation and characterization of *Alb-CreER* mice.
a Schematic diagram showing the knock-in strategy for the *Alb-CreER* mouse line. **b** Schematic diagram showing the experimental design for genetic tracing of Alb^{+} cells. **c** Immunostaining for tdTomato and HNF4α (hepatocyte marker) on liver sections collected from *Alb-CreER;R26-L-tdT* mice treated with Tam. **d** Immunostaining for tdTomato and cell lineage markers CK19, Desmin, or VE-cad. **e** Schematic diagram showing the experimental design to assess Tam-induced removal of the rox-flanked Stop sequence in Tam treated *Alb-CreER;R26-RL-GFP* mice. **f** A schematic diagram showing the experimental design. **g** Immunostaining for GFP, GS, β-catenin, and E-cad on liver sections. **h** Quantification of the percentage of hepatocytes expressing GFP. Data are mean ± SEM; n = 5 mice. Scale bars, 100 μm.

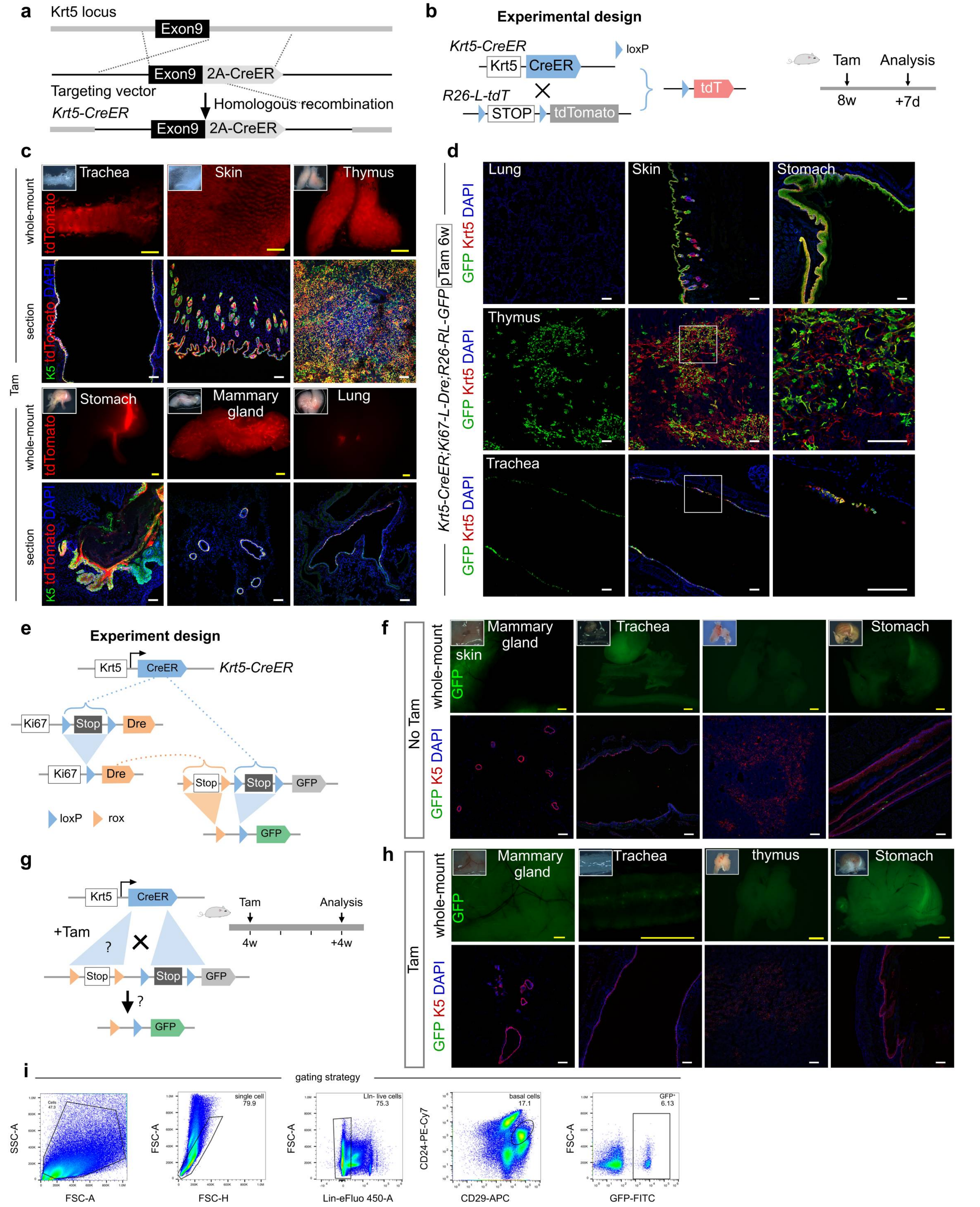

Supplementary Figure 4. Characterization of *Krt5-CreER* mice.

a Schematic diagram showing the knock-in strategy for generation of *Krt5-CreER* mouse line. **b** Schematic figure showing the experimental design of genetic tracing of Krt5^{+} cells. **c** Whole-mount bright-field (insert) and fluorescence images of tissues and organs collected from Tam treated *Krt5-CreER;R26-L-tdT* mice. The lower panel shows images of these tissue sections stained with tdTomato and Krt5 (K5). **d** Immunostaing for Krt5 and GFP on multiple tissue sections collected from *Krt5-CreER;Ki67-L-Dre;R26-RL-GFP* mice 6 weeks post tamoxifen induction. **e** Schematic diagram showing the strategy for tracing Krt5^{+} cell proliferation. **f** Whole-mount bright-field (insert) and fluorescence images, and tissue section staining images of tissues collected from corn oil-treated (no tam) *Krt5-CreER;Ki67-L-Dre;R26-RL-GFP* mice. **g** Schematic diagram showing examination of Cre-rox recombination after Tam treatment in *Krt5-CreER;R26-RL-GFP* mice. **h** Whole-mount bright-field (insert) and fluorescence images, and tissue section staining images of tissues collected from Tam-treated *Krt5-CreER;R26-RL-GFP* mice. **i** Successive gating shows sequential selection of basal cells of FSC and SSC, single cells were gated with FSC and FSH. Live cells were DAPI (eFluo 450) negative. Epithelial cells were stained with lineage-specific (Lin) antibodies against endothelial cells (CD31-eFluor 450), erythroid cells (Ter119-eFluor 450), and hematopoietic cells (CD45-eFluor 450). Yellow bars, 1 mm; white bars, 100 μm.

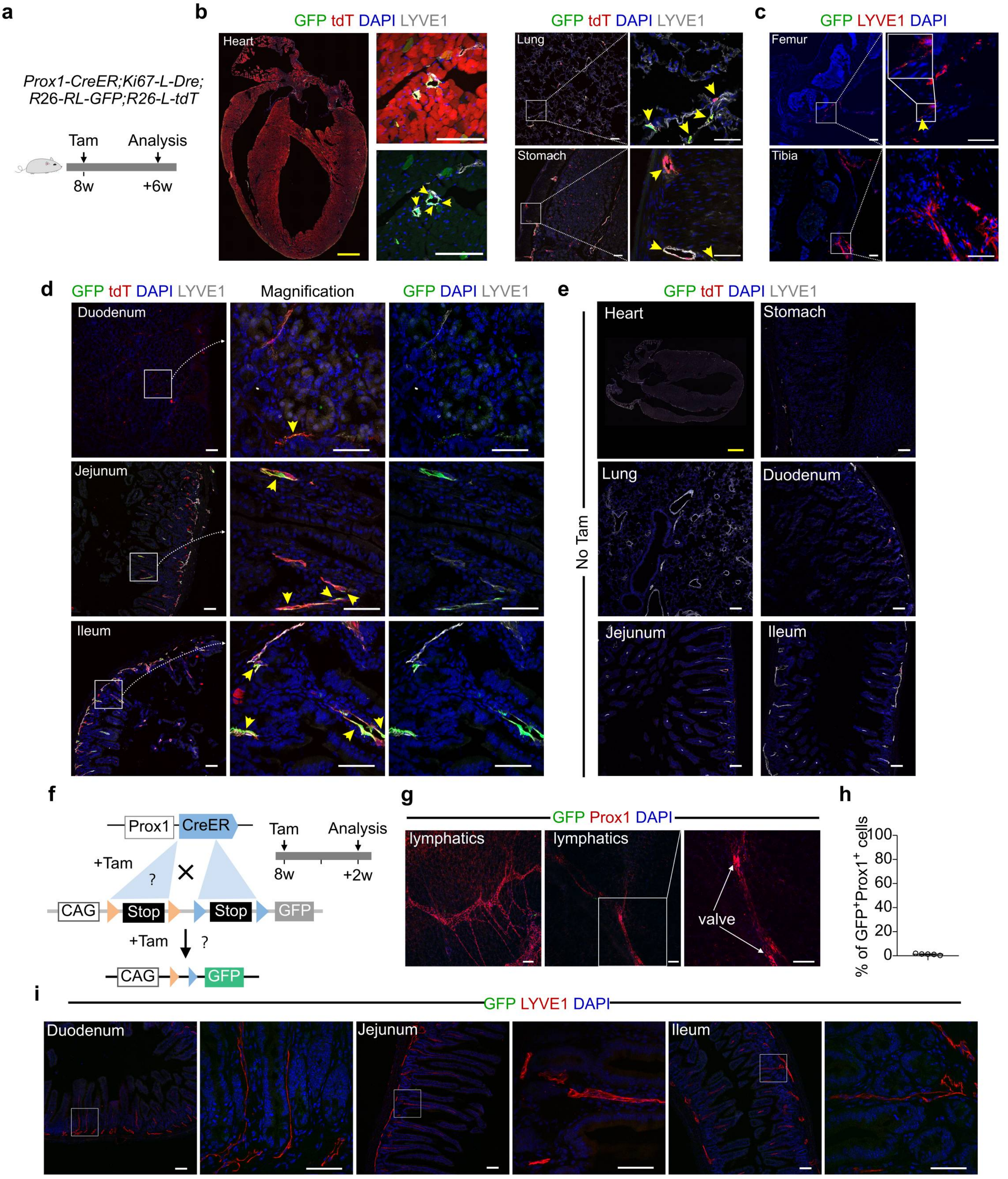

Supplementary Figure 5. Examination of *Prox1-CreER*-labeled cell proliferation in multiple organs.
a Experimental design of tracing Prox1$^+$ cell proliferation in adult fProTracer mice. **b** Immunostaining for GFP, tdT, and LYVE1 on heart, lung, and stomach sections collected from *Prox1-CreER;Ki67-L-Dre;R26-RL-GFP;R26-L-tdT* mice 6 weeks post Tam induction. Arrowheads, GFP$^+$LYVE1$^+$ cells. **c** Immunostaining for GFP and LYVE1 on bone sections collected from *Prox1-CreER;Ki67-L-Dre;R26-RL-GFP* mice 6 weeks post Tam induction. Arrowheads, GFP$^+$LYVE1$^+$ cells. **d** Immunostaining for GFP, tdT, and LYVE1 on intestinal sections collected from *Prox1-CreER;Ki67-L-Dre;R26-RL-GFP;R26-L-tdT* mice 6 weeks post Tam induction. Arrowheads, GFP+tdT$^+$LYVE1$^+$ cells. **e** Immunostaining for GFP, tdT, and LYVE1 on tissue sections collected from corn-oil (no Tam) treated *Prox1-CreER;Ki67-L-Dre;R26-RL-GFP;R26-L-tdT* mice. No GFP signals were detected in tissue sections indicated nearly no leakiness of lymphatic fProTracer. **f** Schematic diagram showing the experimental strategy. **g** Whole-mount fluorescent staining for GFP and Prox1 on mesenteric lymphatics collected from *Prox1-CreER;R26-RL-GFP* mice 2 weeks post Tam induction. **h** Quantification of the percentage of Prox1$^+$ cells expressing GFP. Data are mean ± SEM; n = 5 mice. **i** Immunostaining for GFP and LYVE1 on intestinal sections collected from

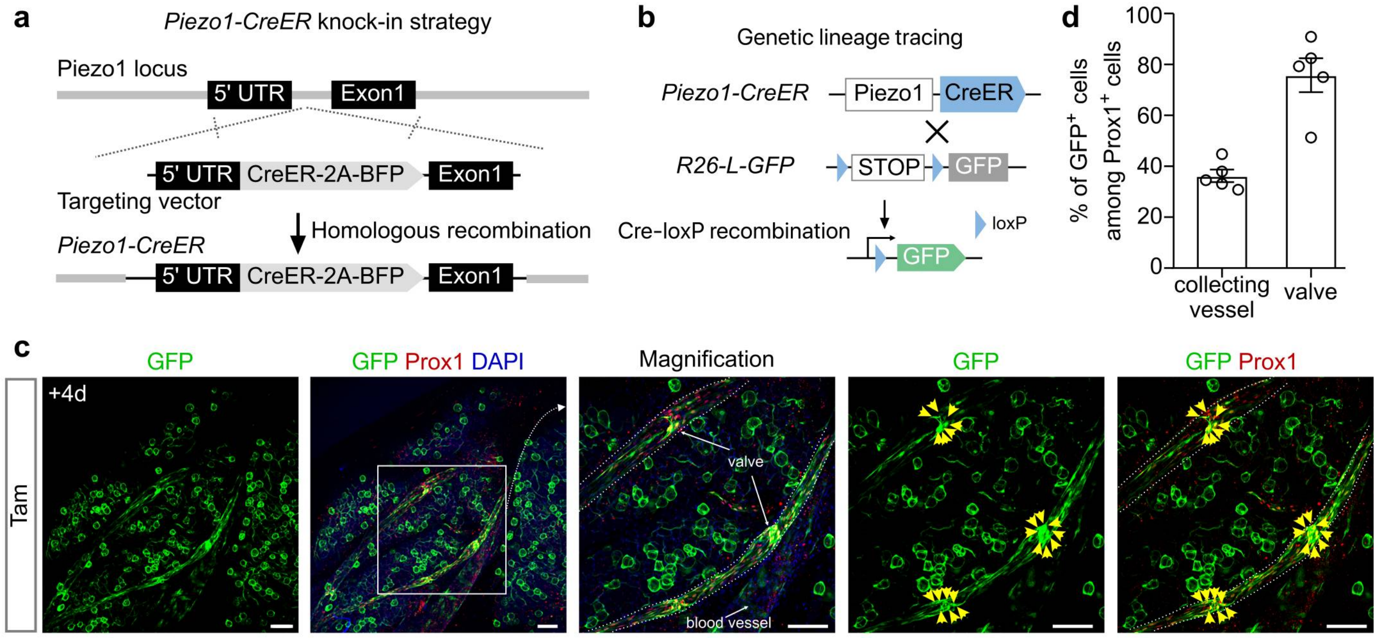

Supplementary Figure 6. Genetic labelling of Piezo1⁺ cells in mesenteric lymphatics.
a The genetic knock-in strategy of *Piezo1-CreER* mouse line. **b** The experimental design of tracing piezo1⁺ cells using *R26-L-GFP* mouse line. **c** Z-stack images of mesenteric lymphatics stained with GFP and Prox1 4 days post tamoxifen induction. Yellow arrowheads indicate GFP⁺ Prox1⁺ cells in valve. **d** Quantification of the labeled cells in different regions of the lymphatics. Scale bars, 100μm.

Supplementary Table 1 Genotyping primers	
Mouse line	Primer sequence
Ki67-L-Dre<br>Mutant (Pr1 and Pr2)<br>Wild type (Pr3 and Pr4)	Pr1: TCAAATGGCTCTCCTCAAGCG<br>Pr2: AGACATCCTCATCAGGGTGTTGTAG<br>Pr3: GGGCTCATTGAGTTGAGGTG<br>Pr4: CCAAAGGAGGCAGCGAATTT
UBC-CreER<br>Mutant (Pr1 and Pr2)	Pr1: TGGATAGTGAAACAGGGGCAATG<br>Pr2: ATAGAGTATGGGGGGCTCAGCATC
R26-RL-GFP<br>Mutant (Pr1 and Pr2)<br>Wild type (Pr3 and Pr4)	Pr1: TAAAAAACCTCCCACACCTCCCCC<br>Pr2: TCACCTTGATGCCGTTCTTCTG<br>Pr3: TTCTGCCTGCTTTTGTCTCCTG<br>Pr4: AGCCTGCCAAGTAACTACTCTTGTG
R26-R-tdT<br>Mutant (Pr1 and Pr2)<br>Wild type (Pr3 and Pr4)	Pr1: ACGGGTGTTGGGTCGTTTGTTC<br>Pr2: TTCTTGTAATCGGGGATGTCGGCG<br>Pr3: AAGGGAGCTGCAGTGGAGTA<br>Pr4: CCGAAAATCTGTGGGAAGTC
CAG-Dre<br>Mutant (Pr1 and Pr2)	Pr1: ACTCCTTGCCGATGTTCCTCAG<br>Pr2: TTGTCCCAAATCTGGCGGAG
ACTB-Cre<br>Mutant (Pr1 and Pr2)	Pr1: CCTGGAAAATGCTTCTGTCCG<br>Pr2: CAGGGTGTTATAAGCAATCCC
Tnni3-Dre<br>Mutant (Pr1 and Pr2)<br>Wild type (Pr1 and Pr3)	Pr1: ATTTAGTCTTTGTCCTCGCCCC<br>Pr2: TACTCCTTGCCGATGTTCCTCAGG<br>Pr3: AGGTTCCCTACCCCACTTCTTAGC
Alb-CreER<br>Mutant (Pr1 and Pr2)<br>Wild type (Pr1 and Pr3)	Pr1: ACCTTTCTCCTCCTCCTCTTCGTC<br>Pr2: TTGGTCAGTAAGCCCATCATCG<br>Pr3: GCAATGGTTCCTCTCTGCTACACTC
R26-L-tdT<br>Mutant (Pr1 and Pr2)<br>Wild type (Pr3 and Pr4)	Pr1: GGCATTAAAGCAGCGTATCC<br>Pr2: CTGTTCCTGTACGGCATGG<br>Pr3: AAGGGAGCTGCAGTGGAGTA<br>Pr4: CCGAAAATCTGTGGGAAGTC
Ktr5-CreER<br>Mutant (Pr1 and Pr2)<br>Wild type (Pr2 and Pr3)	Pr1: CGGGCTCTACTTCATCGCAT<br>Pr2: ACCAAAGCATGTGGTTCTGC<br>Pr3: GTTTTCTGTCAGGGACCCCA
β-cateninfl/+	Pr1: AGAATCACGGTGACCTGGGTTAAA

Mutant (Pr1 and Pr2)	Pr2: CAGCCAAGGAGAGCAGGTGAGG
Prox1-CreER<br>Mutant (Pr1 and Pr3)<br>Wild type (Pr2 and Pr3)	Pr1: TGCTGCTGGCTACATCATCTCG<br>Pr2: ATGTCTCCTCTGAAACCCACGG<br>Pr3: TCCCTTCTCCTGAAAACCAACC
Piezo1$^{fl/+}$<br>Mutant (Pr1 and Pr2)	Pr1: GCCTAGATTCACCTGGCTTC<br>Pr2: GCTCTTAACCATTGAGCCATCT
Vegfr3$^{fl/+}$<br>Mutant (Pr1 and Pr2)	Pr1: AGAGACTTCCTGAGCTGTTTCC<br>Pr2: ACATCGAGTCCTTCCTGTTGAC
Piezo1-CreER<br>Mutant (Pr1 and Pr3)<br>Wild type (Pr1 and Pr2)	Pr1: AATAGAAAGTCGCGCTCCCC<br>Pr2: CGAGCTTATAAAGGCCCGCA<br>Pr3: CCACTCCCACTGTCCTTTCC
