# Supplementary material for: Functional ProTracer identifies patterns of cell proliferation in tissues and underlying regulatory mechanisms
Source: NPJ Regen Med. 2023 Aug 3;8:41. doi: 10.1038/s41536-023-00318-y (PMC10400583; doi:10.1038/s41536-023-00318-y)
Supplement: Supplementary file 2 — Reporting Summary [file 41536_2023_318_MOESM2_ESM.pdf]

Corresponding author(s): Bin Zhou

Last updated by author(s): Jul 17, 2023

Reporting Summary

Nature Portfolio wishes to improve the reproducibility of the work that we publish. This form provides structure for consistency and transparency in reporting. For further information on Nature Portfolio policies, see our Editorial Policies and the Editorial Policy Checklist.

Statistics

For all statistical analyses, confirm that the following items are present in the figure legend, table legend, main text, or Methods section.

n/a	Confirmed	
☐	☒	The exact sample size (*n*) for each experimental group/condition, given as a discrete number and unit of measurement
☐	☒	A statement on whether measurements were taken from distinct samples or whether the same sample was measured repeatedly
☐	☒	The statistical test(s) used AND whether they are one- or two-sided<br>*Only common tests should be described solely by name; describe more complex techniques in the Methods section.*
☒	☐	A description of all covariates tested
☒	☐	A description of any assumptions or corrections, such as tests of normality and adjustment for multiple comparisons
☒	☐	A full description of the statistical parameters including central tendency (e.g. means) or other basic estimates (e.g. regression coefficient) AND variation (e.g. standard deviation) or associated estimates of uncertainty (e.g. confidence intervals)
☒	☐	For null hypothesis testing, the test statistic (e.g. *F*, *t*, *r*) with confidence intervals, effect sizes, degrees of freedom and *P* value noted<br>*Give P values as exact values whenever suitable.*
☒	☐	For Bayesian analysis, information on the choice of priors and Markov chain Monte Carlo settings
☒	☐	For hierarchical and complex designs, identification of the appropriate level for tests and full reporting of outcomes
☒	☐	Estimates of effect sizes (e.g. Cohen's *d*, Pearson's *r*), indicating how they were calculated

Our web collection on statistics for biologists contains articles on many of the points above.

Software and code

Policy information about availability of computer code

Data collection	Zeiss stereoscope (Axio Zoom. V16) was used for whole-mount bright-field and fluorescence image collection. Zeiss LSM880 confocal and Nikon A1 confocal were used for immunofluorescence data collection. Thermo Attune NxT Flow Cytometer was used for FACS data collection. Dolphin Doc Plus was used for PCR product electrophoresis imaging. iPhone 14 Pro was used for the images of mouse appearance.
Data analysis	Fiji (1.0) and Photoline (18.5.1) were used for immunofluorescence and bright-filed image analysis. FlowJo (10.4) was used for FACS data analysis. GraphPad Prism (8.0.0) was used for data analysis.

For manuscripts utilizing custom algorithms or software that are central to the research but not yet described in published literature, software must be made available to editors and reviewers. We strongly encourage code deposition in a community repository (e.g. GitHub). See the Nature Portfolio guidelines for submitting code & software for further information.

Data

Policy information about availability of data

All manuscripts must include a data availability statement. This statement should provide the following information, where applicable:

- Accession codes, unique identifiers, or web links for publicly available datasets
- A description of any restrictions on data availability
- For clinical datasets or third party data, please ensure that the statement adheres to our policy

All data generated by this study are included in this article and its supplementary materials. Source data will be provided upon request.

Research involving human participants, their data, or biological material

Policy information about studies with human participants or human data. See also policy information about sex, gender (identity/presentation), and sexual orientation and race, ethnicity and racism.

Reporting on sex and gender	*Use the terms sex (biological attribute) and gender (shaped by social and cultural circumstances) carefully in order to avoid confusing both terms. Indicate if findings apply to only one sex or gender; describe whether sex and gender were considered in study design; whether sex and/or gender was determined based on self-reporting or assigned and methods used. Provide in the source data disaggregated sex and gender data, where this information has been collected, and if consent has been obtained for sharing of individual-level data; provide overall numbers in this Reporting Summary. Please state if this information has not been collected. Report sex- and gender-based analyses where performed, justify reasons for lack of sex- and gender-based analysis.*
Reporting on race, ethnicity, or other socially relevant groupings	*Please specify the socially constructed or socially relevant categorization variable(s) used in your manuscript and explain why they were used. Please note that such variables should not be used as proxies for other socially constructed/relevant variables (for example, race or ethnicity should not be used as a proxy for socioeconomic status). Provide clear definitions of the relevant terms used, how they were provided (by the participants/respondents, the researchers, or third parties), and the method(s) used to classify people into the different categories (e.g. self-report, census or administrative data, social media data, etc.) Please provide details about how you controlled for confounding variables in your analyses.*
Population characteristics	*Describe the covariate-relevant population characteristics of the human research participants (e.g. age, genotypic information, past and current diagnosis and treatment categories). If you filled out the behavioural & social sciences study design questions and have nothing to add here, write "See above."*
Recruitment	*Describe how participants were recruited. Outline any potential self-selection bias or other biases that may be present and how these are likely to impact results.*
Ethics oversight	*Identify the organization(s) that approved the study protocol.*

Note that full information on the approval of the study protocol must also be provided in the manuscript.

Field-specific reporting

Please select the one below that is the best fit for your research. If you are not sure, read the appropriate sections before making your selection.

☒ Life sciences ☐ Behavioural & social sciences ☐ Ecological, evolutionary & environmental sciences

For a reference copy of the document with all sections, see nature.com/documents/nr-reporting-summary-flat.pdf

Life sciences study design

All studies must disclose on these points even when the disclosure is negative.

Sample size	All experiments were repeated at least three times with similar results. Each sample size was described in detail in each figure legend. No statistical methods were used to predetermine the sample size. A minimun number of animals were used according to standard scientific conventions.
Data exclusions	No data in the experiments was excluded.
Replication	For each animal experiment, at least 3 repeats were done to confirm the reproducibility of the findings. n means biological replicates (number of mice or patients) and is indicated in the manuscript. Replicated experiments yielded reproducible results.
Randomization	For all animal experiments, experimental and control animals were randomly allocated from the appropriated genotype. Sample were allocated randomly to different experimental groups.
Blinding	Different investigators performed the mouse treatment/sacrifice and image acquisition/analysis. The investigator performing the quantitative analysis of the images was blinded to the mouse genotypes.

Reporting for specific materials, systems and methods

We require information from authors about some types of materials, experimental systems and methods used in many studies. Here, indicate whether each material, system or method listed is relevant to your study. If you are not sure if a list item applies to your research, read the appropriate section before selecting a response.

Materials & experimental systems

n/a	Involved in the study
☐	☒ Antibodies
☒	☐ Eukaryotic cell lines
☒	☐ Palaeontology and archaeology
☐	☒ Animals and other organisms
☒	☐ Clinical data
☒	☐ Dual use research of concern
☒	☐ Plants

Methods

n/a	Involved in the study
☒	☐ ChIP-seq
☐	☒ Flow cytometry
☒	☐ MRI-based neuroimaging

Antibodies

Antibodies used

GFP (GF090R, nacalai tesque, 04404-84, 1:500), manufacturer validated by IF for mouse tissue.
tdTomato (Rockland, 600-401-379, 1:500), manufacturer validated by IF for mouse tissue.
β-catenin (BD Pharmingenp, 610153, 1:100), manufacturer validated for A431 cell line by IF.
GS (Abcam, Ab49873, 1:1000), Ma R et al. (2020) validated for human tissue by IHC.
E-cadherin (E-cad, 24E10, Cell signaling, 3195, 1:100), manufacturer validated for human tissue line by IHC.
HNF4a (Cell Signalling, 3113s, 1:500), manufacturer validated by IHC for human tissue.
CK19 (Developmental Studies Hybridoma Bank, TROMA-III, 1:500), manufacturer validated by IHC for mosue tissue.
Krt5 (Covance, 905504, 1:500), Hu Y et al. (2001) validated by IHC for mouse keratinocytes.
Prox1 (Abcam, ab101851), manufacturer validated by IHC for mouse tissue.
PECAM (BD Pharmingen, 553370, 1:500), Suri C et al. (1996) validated by IHC for mouse embryos.
TNNI3 (Abcam, ab56357, 1:200), manufacturer validated by WB for human heart tissue.
Desmin (R&D, AF3844, 1:100), manufacturer validated by IF for mouse skeletal muscle.
VE-cad (R&D, AF1002, 1:100), manufacturer validated by IHC for mouse heart.
LYVE1 (eBioscience, 53-0443-80, 1:250), manufacturer validated by IF for mouse intestine.
DyLight™ 405 AffiniPure Donkey Anti-Rabbit IgG (H+L) (Jackson ImmunoResearch Laboratory Inc., 711-475-152, 1:1000), Yamashita, Y et al. (2017) validated by IF for mouse tissues.
Donkey anti-rabbit IgG (H+L) Highly Cross-Adsorbed Secondary Antibody, Alexa FluorTM 555 (Thermo fisher scientific, A31572, 1:500), manufacturer validated for detection of rabbit IgG on human cell line by ICC/IF.
Donkey anti-rabbit IgG (H+L) Highly Cross-Adsorbed Secondary Antibody, Alexa FluorTM 488 (Thermo fisher scientific, A21206, 1:500), manufacturer validated for detection of rabbit IgG on human cell line by ICC/IF.
Donkey anti-rabbit IgG (H+L) Highly Cross-Adsorbed Secondary Antibody, Alexa FluorTM 647 (Thermo fisher scientific, A31573, 1:500), manufacturer validated for detection of rabbit IgG on human cell line by ICC/IF.
Donkey anti-Goat IgG (H+L) Highly Cross-Adsorbed Secondary Antibody, Alexa FluorTM 555 (Thermo fisher scientific, A21432, 1:500), manufacturer validated for detection of goat IgG on human cell line by ICC/IF.
Donkey anti-Goat IgG (H+L) Highly Cross-Adsorbed Secondary Antibody, Alexa FluorTM 488 (Thermo fisher scientific, A11055, 1:500), manufacturer validated for detection of goat IgG on human cell line by ICC/IF.
Donkey anti-Goat IgG (H+L) Highly Cross-Adsorbed Secondary Antibody, Alexa FluorTM 647 (Thermo fisher scientific, A21447, 1:500), manufacturer validated for detection of goat IgG on human cell line by ICC/IF.
Donkey anti-Rat IgG (H+L) Highly Cross-Adsorbed Secondary Antibody, Alexa FluorTM 488 (Thermo fisher scientific, A21208, 1:500), manufacturer validated for detection of rat IgG on human cell line by ICC/IF.
Donkey anti-Mouse IgG (H+L) Highly Cross-Adsorbed Secondary Antibody, Alexa FluorTM 647 (Thermo fisher scientific, A31571, 1:500), manufacturer validated for detection of mouse IgG on human cell line by ICC/IF.
Immpress HRP horse anti-rabbit IgG Polymer Detection Kit, Peroxidase (Vector laboratories, MP-7401, 1:1), manufacturer validated for mouse tissue by IHC.
Peroxidase AffiniPure Goat Anti-Rabbit IgG (Jackson ImmunoResearch, 111-035-047, 1:4000), Speckmann T et al (2016) validated for mouse cells by western.
Peroxidase AffiniPure Donkey Anti-Mouse IgG (Jackson ImmunoResearch, 715-035-150, 1:4000), Usman W et al (2018) validated for mouse cells by western.

Validation

Validation statements are included in the statement for "Antibodies used" above.

Animals and other research organisms

Policy information about studies involving animals; ARRIVE guidelines recommended for reporting animal research, and Sex and Gender in Research

Laboratory animals

Mice of both male and female at the age of 8-20 weeks were used for experiments with similar aged mice for both control and experimental groups. All mice were maintained on a 129, C57BL6 and ICR mixed background. Prox1-CreER, β-cateninfl/+, Piezo1fl/+, VEGFR3fl/+, UBC-CreER, R26-R-tdT, Tnni3-Dre, ACTB-Cre, CAG-Dre, R26-L-tdT, Piezo1-CreER, Ki67-L-Dre, R26-RL-GFP, Alb-CreER, Krt5-CreER mouse lines were used in this study. All mice were housed at the laboratory Animal Center of the Center for Excellence in Molecular Cell Science in a Specific Pathogen Free (SPF) facility with individually ventilated cages. The room has controlled temperature (20-25°C), humidity (30%-70%) and light (12 hour light-dark cycle). Mice were provided ad libitum access to a regular rodent chow diet.

Wild animals

No wild animals were included in this study.

Reporting on sex	The findings apply to both male and female mice. Mice with the appropriate genotype were randomly allocated to different groups. Approximately equal number of female and male mice were used in each group for analysis.
Field-collected samples	No field-collected samples were included in this study.
Ethics oversight	All mice were used in accordance with the guidelines of the Institutional Animal Care and Use Committee (IACUC) of Shanghai Institute of Biochemistry and Cell Biology, Chinese Academy of Sciences. The animal protocol number is SIBCB-S374-1702-001-C1.

Note that full information on the approval of the study protocol must also be provided in the manuscript.

Flow Cytometry

Plots

Confirm that:

☒ The axis labels state the marker and fluorochrome used (e.g. CD4-FITC).

☒ The axis scales are clearly visible. Include numbers along axes only for bottom left plot of group (a 'group' is an analysis of identical markers).

☒ All plots are contour plots with outliers or pseudocolor plots.

☒ A numerical value for number of cells or percentage (with statistics) is provided.

Methodology

Sample preparation	Briefly, the third and fourth mammary glands (without lymphatic nodes) were collected and minced into small pieces. Then the samples were digested with an enzyme mix (5% fetal bovine serum, 1% penicillin-streptomycin-glutamine, 25 mM HEPES, 3000 U collagenase III (Worthington) dissolved in RPMI 1640 or DMEM) on a rotor at 120 rpm at 37 ? for 90 min. The mix was centrifuged at 1000 rpm for 5 min to discard the supernatant which contained fatty tissue. The collected sediment was treated with red blood cell lysis buffer (eBioscience) at room temperature for 5 min. The incubation was stopped with Hank's Balanced Salt Solution (HBSS) and the sample was centrifuged at 1000 rpm for 4 min to collect the tissues. The tissues were further digested with 0.25% trypsin-EDTA (Invitrogen) at 37 ? for 5 min and the digestion was stopped with the addition of DMEM and 0.1 mg/mL DNase I (Worthington) for a further 5 min. The single-cell suspension was collected by filtering through 70 µm cell strainers. Then cells were incubated with Fc block at room temperature for 5 min. After that, primary antibodies were added to the incubation mix for labeling the mammary cells at 4 ? for 30 min. After washing with HBSS solution, cells were re-suspended with HBSS containing DAPI.
Instrument	Thermo Attune NxT Flow cytometer
Software	FlowJo (10.4)
Cell population abundance	about 2*10^6 mammary gland cells were analyzed. Basal cells comprised about 10% of all cells.
Gating strategy	First, remove small debris in FSC-A verse SSC-A gating. And then doublets were excluded in SSC-A verse SSC-H gating. Dead cells were excluded on DAPI staining. Then basal cell population was determined on the gate of CD24mid/CD29hi antibody staining. Gating strategies are shown in Supplementary Figure 4i

☒ Tick this box to confirm that a figure exemplifying the gating strategy is provided in the Supplementary Information.
